# Supplementary figures and images for: Cerebral Blood Flow in Alzheimer’s Disease: A Meta-Analysis on Transcranial Doppler Investigations
Source: Geriatrics (Basel). 2024 May 4;9(3):58. doi: 10.3390/geriatrics9030058 (PMC11130854; doi:10.3390/geriatrics9030058)

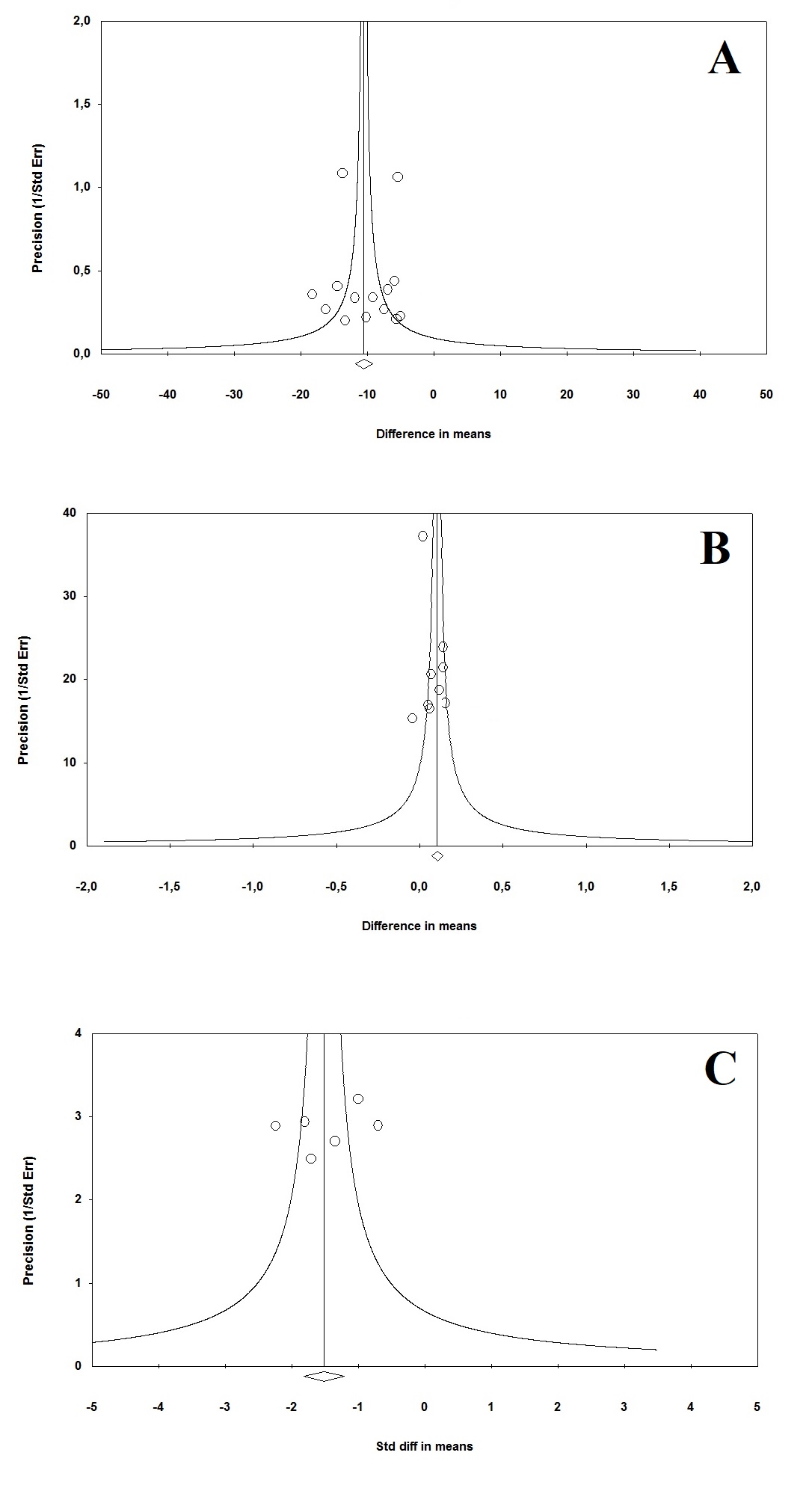

Supplement: Supplementary file 1 [file geriatrics-09-00058-s001.zip › Supplementary file S2.jpg]
